# Social prescribing in nursing home vs community: Experience with doll therapy and cognitive games

**DOI:** 10.1093/geroni/igaf122.2583

**Published:** 2025-12-31

**Authors:** Bobo Hi Po Lau, Rebecca Yung-Choy, Cindy Wong

**Affiliations:** Hong Kong Shue Yan University, North Point, China (People’s Republic); Golden Age Foundation, Wan Chai, Hong Kong; Golden Age Foundation, Wan Chai, Hong Kong

## Abstract

Doll therapy and tablet games are often used among older adults with dementia in nursing homes and in-home rehabilitation. While both therapies may enhance mood and social interactions, doll therapy often targets a cognitively frailer clientele than cognitive games on tablets. In Smart Care Lab 2.0 and 3.0, these therapies were used in Hong Kong nursing homes and in-home rehabilitation respectively. Volunteers were trained to facilitate implementation on-site, while (in)formal caregivers were tasked with dispensing these therapies in their daily care to maximize the desired outcomes. Individual interviews, focus groups, and pre-post intervention assessments were conducted to reveal their efficacy and factors affecting their implementation. Results show that efficacies differ by contexts. For doll therapy, their use in nursing homes primarily enhanced residents’ attachment to daily routines and reduced distress, yet their use in the community fostered social interactions. For cognitive games, their use in nursing homes were restricted by poor dexterity and mobility of the residents, yet their use in the community potentially bridged older adults with cognitive impairment to other technologies for their independent living. Echoing social prescribing, a professional ‘link worker’ is essential for identifying suitable cases, training volunteers and (in)formal caregivers for dispensing the therapy and moderating the intervention clients’ changing capabilities. Through implementing the same therapies to clients in two contexts with similar volunteer training involved, this study provides a unique opportunity to compare the differences in implementation factors to inform the generalization of technologies and tools across care settings.

